# Strontium isotope proxy of sedimentological records reveals uplift and erosion in the Southeastern Neo-Tethys ocean during the late Cretaceous

**DOI:** 10.1038/s41598-024-54128-3

**Published:** 2024-02-12

**Authors:** Amin Navidtalab, Hamzeh Mehrabi, Hadi Shafaii Moghadam, Hossain Rahimpour-Bonab

**Affiliations:** 1 School of Earth Sciences, Damghan University, Damghan, 36716-45667 Iran; 2 School of Geology, College of Science, University of Tehran, Tehran, Iran; 3 Department of Geological Engineering, Karadeniz Technical University, 61100 Trabzon, Turkey

**Keywords:** Solid Earth sciences, Geochemistry, Sedimentology

## Abstract

The mutual interplays between plate tectonic processes, orogenesis and continental uplift, high-flux magmatism, and high sedimentation rates can affect the geochemical signatures and composition of marine sediments. Here, we examine two major disconformities, the Cenomanian–Turonian boundary (CT-ES) and the middle Turonian (mT-ES) exposure surfaces, from the Upper Cretaceous sedimentary successions of the southeastern Neo-Tethys Ocean in the Zagros Basin (Iran). The disconformities are expressed as distinct positive peaks in rubidium (Rb) contents and ^87^Sr/^86^Sr isotopic ratios of carbonates. The ^87^Sr/^86^Sr values of samples bracketing the disconformities in seven well cores give average age ranges of 94.4–93.6 Ma for the CT-ES and 91–86 Ma for the mT-ES. These ages fall in the timespan of forearc/ophiolite formation through subduction initiation in the Neo-Tethys realm (southern margin of Eurasia), high convergence velocities between Arabia and Eurasia, and blueschist exhumation. All these processes involved buckling of the Neo-Tethyan lithosphere, initiating the Neo-Tethys closure and a high erosion rate on the Eurasian margin. The first two mechanisms exerted considerable compressional forces on the adjacent carbonate platforms, reactivated basement faults, and led to the uplift and erosion of the Arabian Plate, which provided the high contents of Rb and the high ^87^Sr/^86^Sr ratios in the carbonates.

## Introduction

The Cretaceous period coincided with significant global changes, including sea-level oscillations, biota speciation, and extinction, intense climate variations, large igneous province (LIPs) magmatism, vigorous tectonic activity, and major changes in continental configuration, and resultant oceanic anoxic events (“*deoxygenation*”)^1–4^. Understanding the timing and tempos of these events—and thus their causes—needs detailed studies on the Cretaceous marine sedimentary rocks, which is the main aim of this paper. The causes of these events can be controlled by either far-field, worldwide, or local tectonic events.

**Figure 1:**
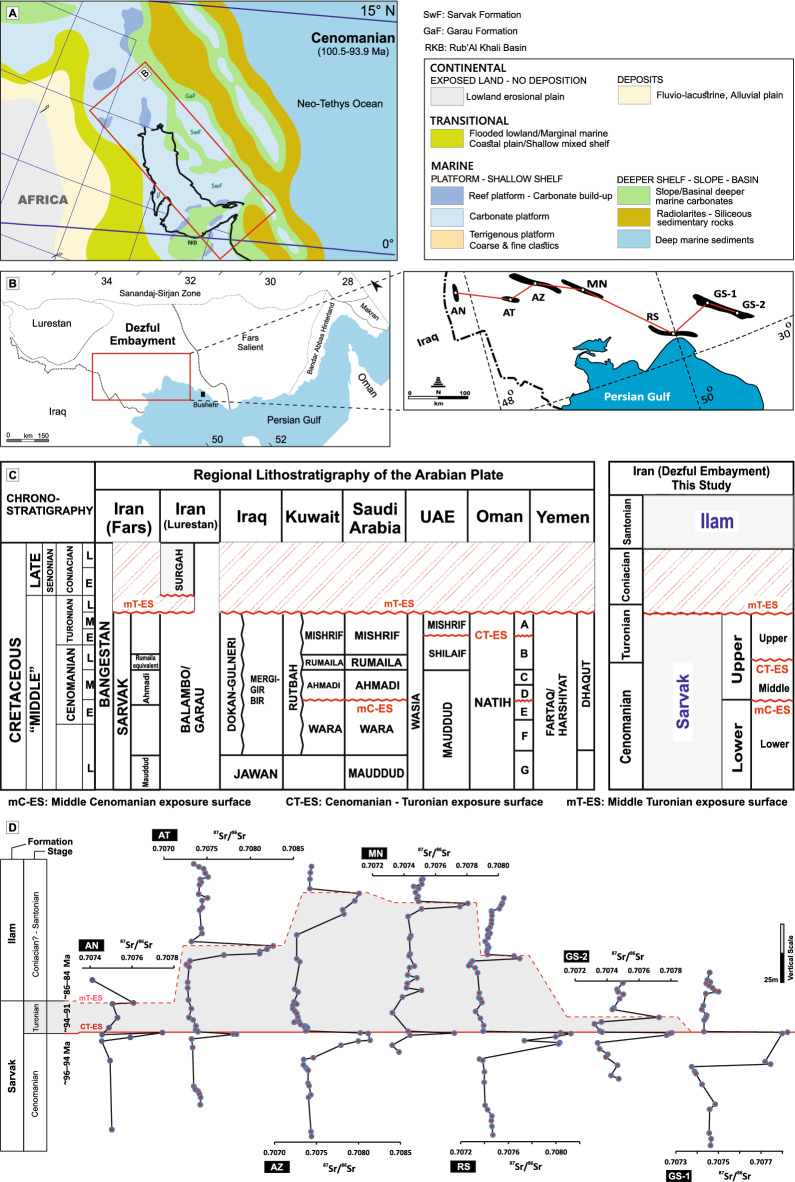
(**A**) Paleogeographic reconstructed map of the southeastern margin of the Neo-Tethys Ocean during the Cenomanian^36^; (**B**) the extension of the Zagros geological subdivisions including the study area (Dezful Embayment); (**C**) Stratigraphic column of the upper Cretaceous rocks in the Middle East and the Dezful Embayment of Zagros Basin in Iran (adopted with some modifications from^75^). (**D**) Rb-uncorrected ^87^Sr/^86^Sr values recorded for well cores along a W-E trend in Zagros showing two positive peaks (except for GS-1 with merged disconformities) associated with the CT-ES and mT-ES disconformities. Cores AZ, MN, and GS-2 are from this study. Core RS is from^22^, cores AN and AT are from^20^, and Core GS-1 is from^21^.

The Late Cretaceous epoch was characterized by an acceleration in plate movements, extensive formation of oceanic crust in the Neo-Tethys Ocean (Fig. 1A), and uplift in the nearby continents along the southern parts of the Mediterranean region, including Cyprus, Turkey, as well as in Caucasus, Iran (Zagros; Fig. 1B) and Oman (i.e., *Bitlis-Zagros suture zone*)^5–11^. The subduction initiation during the middle Cretaceous along the Eurasian (Iranian) margin led to ophiolite formation and extension in the overlying continental crust, which resulted in a remarkable structural reconfiguration of the Zagros area, including the reactivation of basement faults and halokinetic movements^12–17^. The obduction of the Zagros–Oman ophiolites led to further changes in plate kinematics and the closure of oceanic basins in Oman (*Arabian Plate*)^18^. The obduction also changed the rate of subduction of the Neo-Tethyan oceanic lithosphere beneath Eurasia (including Iran and Anatolia, e.g., Agard et al.^19^) and triggered the uplift of the overlying continental crust, leading to enhanced subduction erosion in Zagros^7^. However, there are no detailed studies on the consequence of this continental uplift and the sea-level changes during the Late Cretaceous.

Consequently, several paleo-highs (i.e., *horsts and salt domes*) formed along the Zagros Basin (Fig. 1A-B), where shallow marine carbonate platforms were exposed and eroded^9,20–23^. These exposure events are expressed as three regional disconformities within the Cenomanian–Turonian sequences throughout the Middle East, including the west, south, and southwest of Iran^23–25^, Oman^26,27^, UAE^28,29^, Qatar^30^, Iraq^31,32^, and Turkey^33^. These disconformities represent; (a) the mid-Cenomanian exposure surface (mC-ES), which is traceable throughout the Arabian Plate, (b) the Cenomanian/Turonian boundary exposure surface (CT-ES), which is variably developed and preserved, and (c) the mid-Turonian exposure surface (mT-ES), regionally developed across the Arabian Plate (Fig. 1C).

Earlier discussions have been conducted on the disconformities in the Zagros Basin, covering sedimentological, paleontological, geochemical, and petrophysical aspects^9^. Nevertheless, comprehensive documentation regarding the causal factors, timing, and hiatus durations of these disconformities is lacking and requires further exploration. There are several speculations about the origin and causal factor(s) of these disconformities, including regional uplift (e.g. Berberian and King^10^), ophiolite obduction^22^, peripheral bulging (e.g. Hollis^34^), block faulting and salt diapirism^16^, subduction initiation along the Bitlis-Zagros suture zone (e.g. Maffione et al.^35^), and tectonic inversion^9^.

This study specifically focuses on the cores recovered from oil wells in the Dezful Embayment of the Zagros Basin. The basin was a component of the southeastern Neo-Tethyan carbonate platforms located between paleolatitudes of 0° and 15°N during the Late Cretaceous (Fig. 1A–B)^36^. We isotopically examine the Cenomanian/Turonian (CT-ES) and mid-Turonian (mT-ES) disconformities within the upper Cenomanian–Santonian carbonate successions in a WSW–ESE trend (Fig. 1D) from the southeastern segment of the Zagros Basin of Iran (Fig. 1B). The overall aim is to identify the driving forces behind the formation of CT-ES and mT-ES disconformities documented in the Cenomanian–Santonian carbonate sequences. To accomplish this, we initially assess them through chemo-stratigraphical studies (involving Rb, Sr elemental concentration, and ^87^Sr/^86^Sr isotope ratios), aiming to determine the onset and duration of each associated hiatus. Subsequently, we compare all significant tectonic–magmatic events, such as ophiolite obduction, peripheral bulging, subduction initiation along the Bitlis-Zagros suture zone, and tectonic inversion, along with their respective dating, to the acquired absolute ages of the disconformities. This comparison aims to elucidate the primary causes behind these disconformities. Additionally, it allows us to deduce the driving factor behind the sea-level changes throughout the SE Neo-Tethys Ocean during the Late Cretaceous, as well as the origins of high Rb and ^87^Sr/^86^Sr uptakes.

### Geological setting and stratigraphic background

The Zagros Basin is divided into several structural zones, including the Dezful Embayment—the targeted zone of this study—, Izeh, Lurestan, Fars, and the Persian Gulf (Fig. 1B). The Upper Cretaceous sedimentary sequences in this basin consist of neritic carbonate rocks of the Sarvak and Ilam formations (Cenomanian–Santonian), forming the second most important hydrocarbon reservoir in this region and providing the core materials for this study (Fig. 1B). The studied samples were collected from a west-to-east traverse of oilfields along the southwestern Zagros for Rb elemental content and Sr isotopes analyses (Fig. 1D).

This study includes samples from shallow-water carbonates (ramp-type platforms with predominantly neritic facies and minor pelagic facies), encompassing 361-m thick carbonates in Azadegan (AN), 245-m thick Abteymour carbonates (AT), 275-m thick Ahwaz carbonates (AZ), 145-m thick Marun carbonates (MN), 220-m thick Rag-e Sefid carbonates (RS), 157-m thick Gachsaran 1 (GS-1), and 100-m thick Gachsaran 2 (GS-2) carbonates (Fig. 1B and D). Data for the AN, AT, RS, and GS-1 well cores have been previously published^20–22^, while data for the AZ, MN, and GS-2 cores have been analyzed during this study.

**Figure 2:**
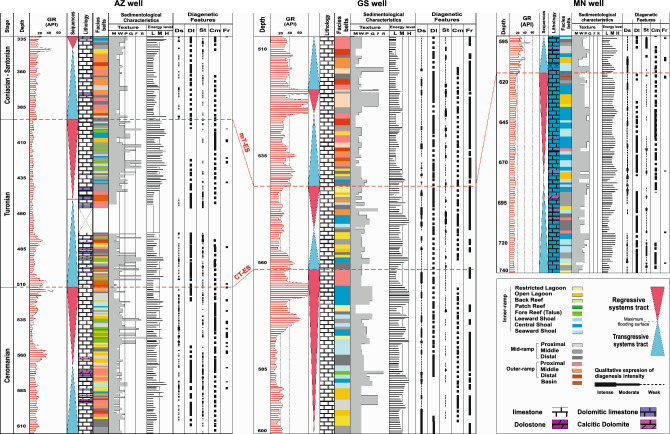
Sedimentological logs of the studied cores AZ, GS-2, and MN showing facies associations and depositional environment, diagenetic features, and Gamma ray (GR) logs. The mid-Turonian (mT-ES) and Cenomanian–Turonian boundary (CT-ES) disconformities are shown. Diagenetic features include Ds: dissolution; Dl: dolomitization; St: staining; Cm: cementation; Fr: fracturing.

**Figure 3:**
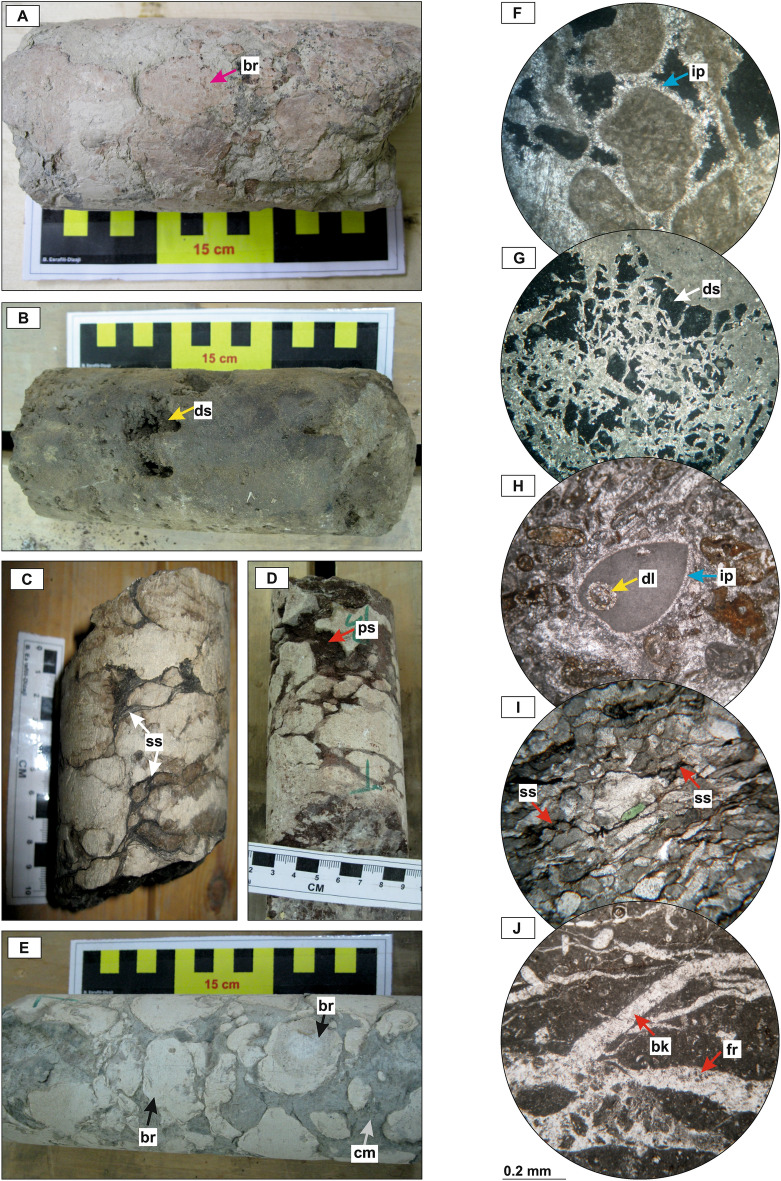
Core photos and photomicrographs of major diagenetic alterations of the Sarvak and Ilam formations in the studied wells. (**A**): brecciation (br); (**B**, **G**): dissolution (ds); (**C**), I: stylolite and solution seams (ss); (**D**): paleosol (ps); (**E**): brecciation (br) and clay-rich matrix (cm) between the breccias; (**F**): isopachous cementation (ip); (**H**): dolomitization (dl) and isopachous cementation (ip); (**J**): fracturing (fr) and blocky calcite cementation (bk). Photos F and G in XPL, H to J in PPL.

Sedimentological logs of these three new wells are presented in Fig. 2. The total studied interval consists of three third-order sequences of Cenomanian (Sv_Cen), Turonian (Sv_Tur), and Coniacian?–Santonian (Il_San) ages. Each sequence comprises open marine (middle ramp, outer ramp, and basin) facies in the TST (transgressive systems tract) and inner ramp (lagoon, shoal, and reef-talus) facies in the RST (regressive systems tract). A general ramp-like carbonate platform model was proposed for these sequences in the Dezful Embayment^33^. The diagenetic history of these sequences encompasses alterations in marine (micritization, bioturbation, isopachous marine cementation; Fig. 3), two stages of meteoric (dissolution/karstification, collapse brecciation, silicification, paleosol formation, meteoric drusy and bladed calcite cementation; Fig. 3), and shallow to deep burial (mechanical to chemical compaction, blocky calcite cementation, stylolite-related dolomitization, recrystallization, pyritization, and fracturing; Fig. 3) realms^22^.

**Figure 4:**
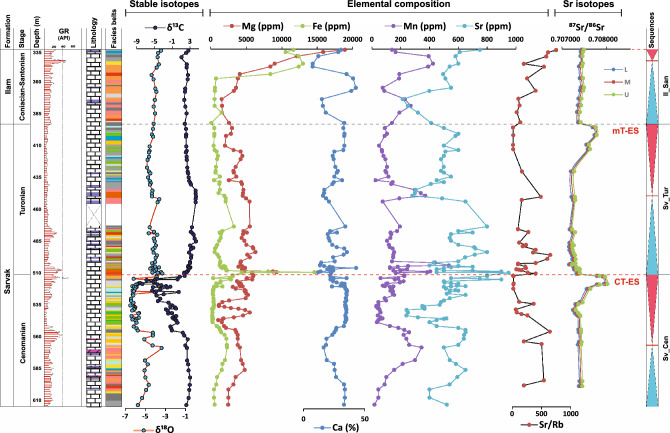
Carbon and oxygen stable isotopes, elemental compositions^23^, Rb-corrected ^87^Sr/^86^Sr ratios, and Rb elemental data of the Sarvak and Ilam formations in well AZ. Rb elemental data are from this study. For lithology and facies pattern, please refer to Fig. 2.

**Figure 5:**
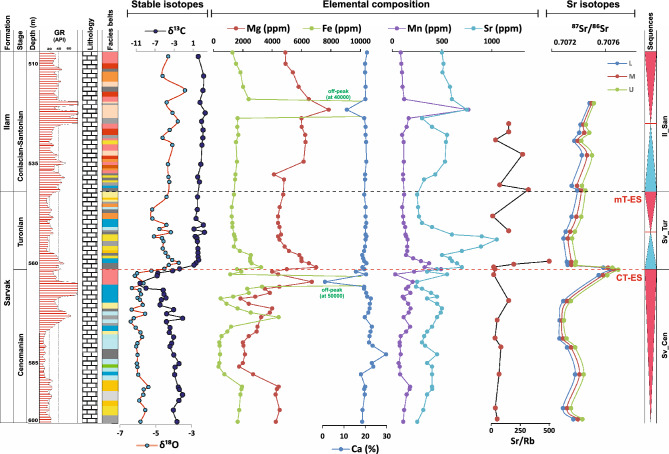
Carbon and oxygen stable isotopes, elemental compositions^23^, Rb-corrected ^87^Sr/^86^Sr ratios, and Rb elemental data of the Sarvak and Ilam formations in well GS-2. For lithology and facies pattern, please refer to Fig. 2.

**Figure 6:**
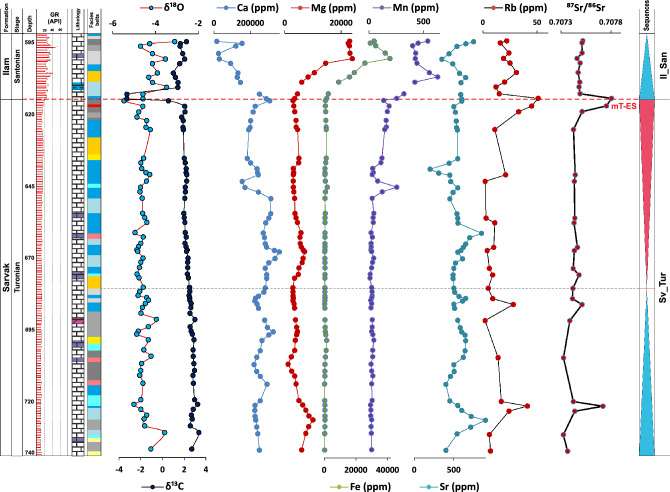
Carbon and oxygen stable isotopes, elemental compositions^23^, Rb-corrected ^87^Sr/^86^Sr ratios, and Rb elemental data of the Sarvak and Ilam formations in well MN. Rb elemental data are also from this study. For lithology and facies pattern, please refer to Fig. 2.

Geochemical logs of the studied cores are presented in Figs. 4, 5 and 6 for the AZ, GS-2, and MN wells, respectively. As shown in these logs, the CT-ES occurs on the top of inner- to proximal outer-ramp facies and is ascribed to the latest Cenomanian. The mT-ES, however, shows variable underlying facies due to irregular erosion and duration^9,20–22^. Due to the lack of precise isotopic data, these two exposure surfaces were considered as a single disconformity in earlier studies (e.g. Hollis^34^, Hajikazemi et al.^37^). However, detailed geochemical-isotopic studies^23,25,38^ have revealed that these are two different exposure surfaces but were merged as a result of severe deep-reaching diagenesis and erosion in some cases. The CT-ES is characterized by younger karstification processes and a short-duration hiatus (< 1 myr) along with very negative δ^13^C and δ^18^O values (to − 10.0 ‰ and – 7.0 ‰, respectively), high Rb contents, and high radiogenic ^87^Sr/^86^Sr ratios. The mT-ES has a similar expression but with mature karstification, mostly higher ^87^Sr/^86^Sr ratios, and quite a long (> 3 myr) hiatus^20–23^.

## Methods

Due to monotonous neritic deposits, sample collection for microfacies and geochemical analyses was in the range of ~ 3 m along the stratigraphic drilled cores. However, it decreased to ~ 0.5 m around the karstified intervals of the paleo-exposure surfaces. We have collected 524 samples for petrographic study and 100 samples for geochemical analyses (*Sr isotopes and Rb elemental content*) from the newly studied cores (*AZ, MN, and GS-2*). Here, we also describe our previous Sr isotope data for some cross sections including AN, AT, RS, and GS-1.

We applied the method developed by Navidtalab et al.^22^ for Sr isotope stratigraphy (SIS) of disconformities in carbonates. The most fine-grained portion of carbonate samples (bulk rock) was powdered with a handheld micro-drill and measured for their ^87^Sr/^86^Sr ratios (see Table 1 in supplementary data) via Thermo Scientific TRITON thermal ionization mass spectrometer (TIMS) at the Boston TIMS Facility, USA. Rubidium (Rb) content was measured using isotope dilution (s.d. 0.3%) at Boston University, USA.

Strontium was extracted through applying standard ion-exchange methods, for loading on rhenium filaments. Isotopic fractionation was corrected through normalizing results to ^87^Sr/^86^Sr = 0.11940. Any inter-laboratory bias corrections were carried out for the samples since a value of 0.7102248 ± 0.000014 (2 s.e., n = 11) was presented by the utilized standard SRM (NIST) 987^39^. Constants and equations introduced by Faure and Mensing^40^ were applied to correct the ^87^Sr/^86^Sr values of the analyzed samples for in-situ decay of Rb elemental content delivered by clay minerals^41^.

The precise stratigraphic position of disconformities and the duration of their associated hiatuses were assessed by applying the method developed by Navidtalab et al.^22^, through which bulk carbonates were sampled by excluding conspicuous diagenetic products including stylolites, fractures, veins, filling cements, and large bioclasts. Then, the ^87^Sr/^86^Sr log was produced for each well core. To determine the numerical age of the strata and evaluate the durations of any hiatus, the selection of the analyzed samples was performed by considering the following criteria: (a) diagenetic examinations to avoid the inclusion of diagenetically altered samples; (b) utilizing δ^13^C values within the range of 0–6‰ and δ^18^O within the range of − 4.5–1‰, respecting the isotopic composition of early Late Cretaceous shallow water carbonates^42–44^; (c) considering elemental contents such as Mg < 100 ppm, Sr > 800 ppm^41,45–47^, Mn < 225 ppm, Fe < 1200 ppm, and Rb ≤ 2 ppm, indicating less-altered samples^22^; and (d) ensuring Sr/Rb ratios are ≥ 200 to indicate minimal incorporation of ^87^Sr through ^87^Rb decay^22,41,48^.

Regarding the uncertainties arising from analytical error and those indicated by the Look-up Table (1σ mean), the calculated mean numerical ages of the analyzed samples depict an uncertainty of ± 1.65 Ma. These age determinations find additional support from biostratigraphic studies, with the Geological Time Scale 2020^49^ serving as the reference for defining stage boundaries. To refine the estimation of hiatal duration, consideration is given to the altered sediments situated between the reliable samples that provide numerical ages. The sedimentation rate of the limestone strata is factored in to calculate the time span of the altered carbonates. Subsequently, the calculated time derived from numerical ages is subtracted by the time span acquired from the sedimentation rate, yielding the duration of hiatus^22^.

## Results

### Core Azadegan (AN)

The ^87^Sr/^86^Sr ratios of the analyzed samples vary between 0.7074106 ± 12 × 10^–6^ to 0.7077455 ± 20 × 10^–6^, with two positive peaks at CT-ES (742–734 m) and mT-ES (691–683 m). The overall Rb concentration fluctuates from 35 to 45 ppm.

### Core Abteymour (AT)

The ^87^Sr/^86^Sr ratios in core AT range from 0.707262 ± 34 × 10^–6^ to 0.708266 ± 38 × 10^–6^. The CT-ES and mT-ES in this section, are represented by two positive peaks around 298.7–291.9 m and 242.5–225 m depth, respectively. For the altered samples, the ^87^Sr/^86^Sr ratios vary between 0.707803 ± 24 × 10^–6^ and 0.707841 ± 38 × 10^–6^, just beneath the CT-ES, and range from 0.707695 ± 24 × 10^–6^ to 0.708266 ± 38 × 10^–6^ beneath the mT-ES. Rb varies between 0 and 42 ppm with two positive peaks (CT-ES: 33–42 ppm; mT-ES: 1–9 ppm), mirroring the intervals defined by the Sr-isotope ratios; both variations show the exposed surfaces.

### Core Ahwaz (AZ)

Sr-isotope ratios of samples derived from core AZ range from 0.707218 ± 38 × 10^–6^ to 0.708138 ± 38 × 10^–6^ (Fig. 1; Supplementary data). Two positive ^87^Sr/^86^Sr isotopic peaks at 512 m and 391 m of the core AZ confirm the exact stratigraphic position of the CT-ES (512 m) and mT-ES (391 m) (Fig. 1). In the lithological units below both disconformities, a gradual upward increase in ^87^Sr/^86^Sr ratios is observed, and an abrupt shift to lower ^87^Sr/^86^Sr ratios above the paleo-exposure surfaces. The altered samples beneath the CT-ES indicate the ^87^Sr/^86^Sr ratios between 0.707462 ± 38 × 10^–6^ and 0.708138 ± 38 × 10^–6^, while below the mT-ES, range from 0.707812 ± 30 × 10^–6^ to 0.708006 ± 36 × 10^–6^. Rb content varies between 0 and 36 ppm with two conspicuous increasing trends (CT-ES: 4–32 ppm; mT-ES: 29–36 ppm), which in their stratigraphic positions match the peaks of the ^87^Sr/^86^Sr ratios.

### Core Marun (MN)

In Core MN, ^87^Sr/^86^Sr ratios of the analyzed samples vary between 0.707325 ± 28 × 10^–6^ and 0.707806 ± 22 × 10^–6^ (Fig. 1; Supplementary data). Two incremental peaks are observed in the ^87^Sr/^86^Sr ratios at 722 m and 613 m depth, which respectively depict the CT-ES and mT-ES. Stratigraphically, the increase in Rb content also follows these two peaks at 722 and 613 m. Rb minimum and maximum values along the section are 2 ppm and 51 ppm, respectively. Beneath both disconformities, upward increasing trends are observed in ^87^Sr/^86^Sr ratios and Rb contents, with a sudden decrease above the paleo-exposure surfaces.

### Core Rag-e Sefid (RS)

The ^87^Sr/^86^Sr ratios from samples of the core RS vary between 0.707268 ± 30 × 10^–6^ and 0.708126 ± 26 × 10^–6^, which record two prominent positive peaks around 746–723 m and 661–657 m, showing the CT-ES and mT-ES, respectively. Samples lying immediately below the CT-ES indicate the ^87^Sr/^86^Sr values between 0.707734 ± 32 × 10^–6^ and 0.708126 ± 26 × 10^–6^, and beneath the mT-ES show values from 0.707648 ± 22 to 0.707696 ± 38. In the same intervals, Rb also shows positive peaks (CT-ES: 25–51 ppm; mT-ES: 29–31 ppm). The Rb content varies between 0 and 51 ppm.

### Core Gachsaran 1 (GS-1)

In this core, the ^87^Sr/^86^Sr ratios range between 0.707375 ± 28 × 10^–6^ and 0.707824 ± 28 × 10^–6^ (Fig. 1). The CT-ES and mT-ES intervals in this core are merged and are expressed through a broad positive peak from 551 to 522 m. The ^87^Sr/^86^Sr ratios of the altered samples vary between 0.707719 ± 26 × 10^–6^ and 0.707824 ± 28 × 10^–6^ immediately beneath the merged surface. The Rb content varies between 0 and 41 ppm and shows a positive peak in the same interval. Rb values of the altered samples below the disconformity range from 32 to 41 ppm.

### Core Gachsaran 2 (GS-2)

In Core GS-2, the ^87^Sr/^86^Sr ratios vary between the minimum of 0.707321 ± 32 × 10^–6^ and the maximum of 0.707805 ± 34 × 10^–6^ (Fig. 1; Supplementary data). CT-ES and mT-ES are characterized by two positive anomalies in ^87^Sr/^86^Sr ratios at stratigraphic positions around 560 m and 545 m depth, respectively. Samples underlying the CT-ES indicate the ^87^Sr/^86^Sr values from 0.707771 ± 32 × 10^–6^ to 0.707805 ± 34 × 10^–6^, and underlying the mT-ES show value of 0.707725 ± 36 × 10^–6^. The Rb content of this core shows a maximum of 29 ppm with two prominent positive peaks. In their stratigraphic position, the Rb peaks follow the positive ^87^Sr/^86^Sr anomalies (CT-ES: 21–29 ppm; mT-ES: 32 ppm). Below both disconformities, a gradual upward increase is observed in the ^87^Sr/^86^Sr ratio and Rb content. Above the paleo-exposure surfaces, they abruptly decrease to significantly lower values.

## Discussion

### Disconformity-related ^87^Sr/^86^Sr ratios

The ^87^Sr/^86^Sr ratios of seawater in the Cenomanian-Santonian interval are expected to vary between a minimum of 0.707284 and a maximum of 0.707446 (Look-Up Table Version 4: 08/ 03)^50,51^. The ^87^Sr/^86^Sr ratios of the studied intervals mostly fall between these end-members, except for those associated with the disconformities (Fig. 1). The ^87^Sr and ^86^Sr isotopes originate from separate sources ^52^; ^86^Sr is non-radiogenic, but ^87^Sr is radiogenic and is generated by the radioactive decay of ^87^Rb. The continental crust has high Rb content because Rb is highly incompatible and tends to be concentrated in magmas during fractional crystallization. Rb^+^ can easily substitute for K in K-feldspar, mica, amphibole, and clay minerals, which are abundant in sialic continental crust rocks (*especially upper crust*) and their alteration products. In contrast, mantle rocks and their partial melts are depleted in Rb. These differences led to ^87^Sr/^86^Sr ~  > 0.708 to 0.716 for the upper continental crust and < 0.705 for Mid-oceanic Ridge Basalt (MORB-)-like mantle melts^53^. The ^87^Sr/^86^Sr ratio of seawater and associated marine carbonates over Earth’s history has been controlled by the interplay between mantle melts and uptake from the continental crust as a result of the weathering of continental sialic rocks during uplift processes *versus* outpouring of mantle-derived basaltic melts in mid-oceanic ridges and subduction zones^54^. Accordingly, any uptake from riverine sediments originating from the continental upper crust could increase the ^87^Sr/^86^Sr ratio of ocean waters. The increased ^87^Sr/^86^Sr ratio at the CT-ES and mT-ES disconformities, therefore, is assigned to the incorporation of ^87^Sr from the continental upper crust through higher rates of riverine flux into the shallow marine environment. The most increased continental flux is achieved during orogenesis and probably during active continental magmatism, while the highest mantle flux is achieved during periods of rapid sea-floor spreading^53^. This suggests that a higher ^87^Sr/^86^Sr ratio at the disconformities could indicate a phase of orogenesis or at least an uplift related to the epeirogeny^55^. More specifically, considering that the ^87^Sr/^86^Sr ratio of the CT-ES and mT-ES falls in the range of the seawater signature (0.706–0.709), the recorded maxima of ^87^Sr/^86^Sr ratios, including 0.708138 in AZ, 0.707806 in MN, and 0.707805 in GS-2 may reflect the uplift of marine limestones^55^. This better supports the weathering of marine limestones rather than the weathering of old continental shields, which could produce much higher ^87^Sr/^86^Sr values. This is consistent with a phase of regional uplift that has been suggested for the Turonian time (mT-ES) throughout the southeastern Neo-Tethyan margin^10^.

Based on the long-term ^87^Sr/^86^Sr curves, two negative excursions are expected for the Cretaceous, specifically in the Aptian-Albian and Cenomanian-Santonian time intervals^56^, with the latter simultaneous to the focused time interval of this study. Conversely, our data reveal two remarkably positive excursions throughout Cenomanian-Santonian time. Detailed examinations of the ^87^Sr/^8^6Sr isotopic ratios linked to OAE2 indicate a short-term positive excursions in values, particularly during the initial development of OAE2 (the Cenomanian–Turonian boundary), peaking at 0.70747^46,56^. This brief positive excursion was attributed to climatic changes provoked by OAE2 and associated riverine strontium fluxes^46^. In our study, this time corresponds to the CT-ES; however, ^87^Sr/^86^Sr values vary between 0.70780 (in core AT) and 0.70802 (in core AZ), which are considerably higher. Moreover, for the Turonian stage, a completely plunging trend is observed worldwide, attributed to the breaking marine water stratification and incorporation of large volume of hydrothermal strontium^46,56^. However, our data indicate another very positive excursion for this time (mT-ES: between 0.70772 in GS-2 and 0.70826 in AT). Since the seawater Sr-isotope curve is considered as a global record of the entire oceans^57^, any variations in the curve necessitate globally impactful alterations in riverine or hydrothermal sources. Therefore, these comparisons dissociate the very positive values in the studied region from global climates and sea-level fluctuations, and they might be explained by regional triggers. Nonetheless, the increase in ^87^Sr/^86^Sr values associated with the CT-ES could be partially, but not entirely, attributed to the global alterations.

A phase of regional uplift has been suggested for the Turonian time (mT-ES) throughout the southeastern Neo-Tethyan margin, which supports the idea of regional rather than the global alteration^10^. Diagenetic products beneath the mT-ES, including mature karstification features such as large-scale dissolution, solution-collapsed breccias, and weathered limestone nodules in a clayey matrix (paleosol) further confirm uplift. Similar products are also observed at the CT-ES, indicating a younger karstification stage^20–23^. However, the role of continental runoff cannot be neglected because, in addition to the ^87^Sr/^86^Sr ratio, the Rb content considerably increases at the disconformities. The increased Rb could explain the elevated ^87^Sr/^86^Sr ratio at these disconformities and their relations to the neighboring continent's uplifting and erosion. However, that assumption is only true if there has been enough time for the ingrowth of ^87^Sr after deposition, through the ^87^Rb decay.

### Numerical ages of disconformities

**Figure 7:**
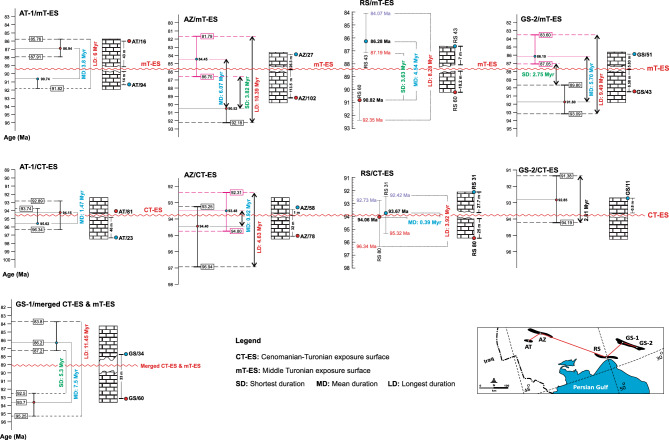
Numerical ages of the samples bracketing the mT-ES and CT-ES disconformities derived from Rb-corrected ^87^Sr/^86^Sr values of the samples. Cores AZ, MN, and GS-2 are from this study. Core RS is from^22^, cores AN and AT are from^20^, and Core GS-1 is from^21^.

To understand the duration of hiatuses associated with disconformities, the Rb-corrected ^87^Sr/^86^Sr of reliable samples bracketing the desired disconformity are converted to the ages according to the Look-Up Table (Version 4: 08/ 03) (Fig. 7)^50,51^. Their derived numerical ages result in a duration time for the hiatus (Timespan A). However, the selected samples are not directly located beneath and over the disconformity due to the presence of a diagenetically altered interval of limestones in between. The altered interval also represents a timespan for deposition which is calculated based on its thickness and the accumulation rates (Timespan B). Therefore, the exact hiatus duration is calculated by subtracting “Timespan B” from “Timespan A”. The commencement of the hiatus is calculated by subtracting the numerical age of the selected sample beneath the disconformity by the calculated deposition timespan of the altered limestones occurring between the sample and the disconformity surface (*for detailed methodology refer to*^22^). Samples confining the CT-ES in Core AZ provide numerical age estimates between 94.4 ± 1.8 and 93.5 ± 1.3 Ma (Fig. 7). In Core GS-2, a sample just above the CT-ES presents an age of 92.9 ± 1.4 Ma. Samples from a sedimentary succession of core AT– enclosing the CT-ES– show age ranges between 95.6 ± 1.9 and 94.2 ± 2.2 Ma^21^, whereas they show ages of 94.0 ± 1.8 and 93.7 ± 1.5 Ma in Core RS^22^. This means the CT-ES is not older than 95.6 Ma (*average: 94.4*) and not younger than 92.9 Ma (*average: 93.6*). Regarding the altered rocks bracketed by the reliable samples (see^22^ for calculation procedure), the hiatus duration associated with the CT-ES is ~ 0.4 m.yr.

Numerical age estimates for samples from lower and upper parts of the mT-ES are between 90.5 ± 1.7 and 84.5 ± 2.5 Ma in Core AZ and between 91.8 ± 1.6 and 86.1 ± 1.7 Ma in Core GS-2. In addition, samples that surround the mT-ES in Core AT provide age estimates ranging between 90.7 ± 1.1 and 86.9 ± 1.1 Ma^21^ and between 90.8 ± 1.6 and 86.3 ± 1.6 Ma in Core RS^22^. The oldest age defined for the mT-ES is 91.8 Ma (*average: 91.0*), whereas the youngest age is 84.5 Ma (*average: 86.0*). By considering the altered rocks confined by the reliable samples, the hiatus duration of the mT-ES is ~ 4.0 m.yr.

Core GS-1 represents a merged CT-ES and mT-ES. Its lower parts show an age of 93.7 Ma, whereas its upper parts have an age of 86.2 Ma. The lower and upper ages perfectly match the lower age of the CT-ES and upper age of the mT-ES, respectively. Regarding these ages and the altered interval, a hiatus duration of ~ 7.45 m.yr. is calculated for this merged disconformity^21^. Although the Sr-isotope data from Core MN depict the disconformities, we do not include them for calculating numerical ages because they come from core cuttings and are not very robust and reliable.

### Correlation with Middle-Late Cretaceous Zagros tectono-magmatic events

Two issues should be considered for understanding the causes of the geochemical disconformities we report in this study. First, what was the cause of the uplift or the sea-level changes during the Late Cretaceous? Second, what were the sources of high Rb and ^87^Sr/^86^Sr uptakes? Although a slight sea-level fall was recorded at about 93.8 Ma around the Cenomanian–Turonian Boundary (CTB)^58^, a regional-scale CT-ES counterpart has not been reported from the central/northwestern Neo-Tethys Ocean. Therefore, the CT-ES disconformity is not solely the product of eustatic sea-level drop. Tectonic inversion has been suggested as the leading cause of the CT-ES in Zagros^9^. The CT-ES and mT-ES disconformities might express the flexural bulging and associated uplift along the Zagros basin of the southeastern Neo-Tethys Ocean. This uplift was also recorded in Turkey and Oman during the Late Cretaceous time and is suggested to be the result of intra-oceanic thrusting and the beginning of oceanic crust emplacement^59–62^. A late Turonian age (90 Ma) was also proposed for the “obduction” related to the Turonian regional unconformity (mT-ES)^11,63^.

**Figure 8:**
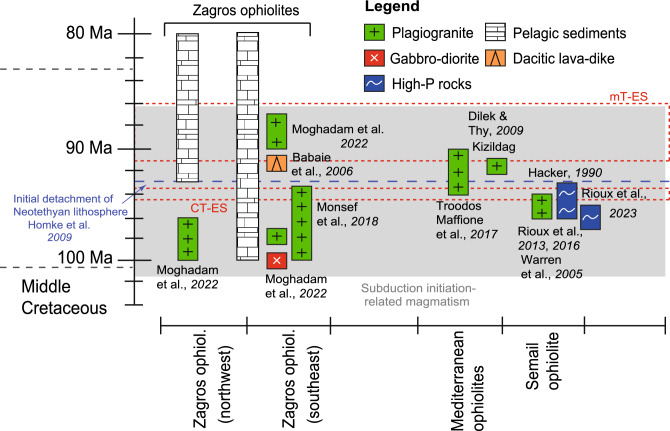
A schematic chart illustrating the chronological sequence of magmatic, high-pressure metamorphic, and sedimentary rocks found in the Zagros, Oman, and Mediterranean ophiolites. Additionally, the chart highlights the occurrences of CT-ES and mT-ES events, along with the initial detachment (intra-oceanic thrusting) of the Neo-Tethyan lithosphere. Please refer to the accompanying text for relevant references.

Subduction initiation and nascent arc formation in the southern Neo-Tethys Ocean have been proposed as responsible for the forearc crust/ophiolite formation during the middle to Late Cretaceous along the Bitlis-Zagros suture zone. These Neo-Tethyan ophiolites have nearly the same age: zircon U–Pb ages of 94–90 Ma for Troodos plagiogranites^35^; 96–95 Ma for Samail ophiolite gabbros and plagiogranites^64–67^; 92–91 Ma for Kizildag plagiogranites^18^ and 100–96 Ma for Zagros ophiolites^68^ (Fig. 8). The subduction initiation was first generated the forearc oceanic crust and a nascent arc during the subduction of the old Neo-Tethyan oceanic crust beneath the Eurasian plate. The initiation of this subduction triggered the rapid exhumation of the blueschists in Zagros at ~ 70–86 Ma through the plate rollback^69,70^. The exhumation of blueschists is also assumed to be associated with higher convergence velocities, plate acceleration, and Oman ophiolite obduction. This also led to oceanic lithospheric buckling^19^ in the weak zones during Late Cretaceous, and their timing is consistent with the disconformities we report in this study. Moreover, the age of initial thrusting of the Neo-Tethys Ocean is suggested to occur around 93 Ma^62^ which is close to the cooling age of the metamorphic sole of the Oman ophiolite (94.5 Ma)^71^. However, new zircon ages on the Oman metamorphic soles— which could be related to the ophiolite obduction—bracket the timing of prograde garnet and zircon growth in the highest-grade rocks of the metamorphic sole between 96.7 ± 0.1 and 95.2 ± 0.1 Ma and are suggested to overlap with the growth of the overlying ophiolite crust at 96.1 to 95.2 Ma^72^. These ages are similar to the SIS-determined age of the CT-ES, which ranges between 94.4 and 93.6 Ma (Fig. 8). Overall, both disconformities fall in the periods of subduction initiation and exhumation of high-pressure rocks, resulting in compressional forces by tectonic inversion around the CTB in the southeastern Neo-Tethys, which triggered the initial phases of the oceanic closure^9^. This process is assumed to cause a peripheral bulging and associated uplift^34^. The reason for the two-step (CT-ES and mT-ES) disconformity in the Upper Cretaceous sedimentary strata could be ascribed to the impact of ophiolite formation in two or more localities.

However, although lithosphere buckling, ophiolite obduction, and/or tectonic inversion have been the mechanisms of the uplift and disconformities, the high uptake of Rb and ^87^Sr/^86^Sr is not due to these processes. Oceanic crust generation within the forearcs will produce magmatic rocks with low Rb and less radiogenic ^87^Sr/^86^Sr ratios of ~ 0.702 to 0.703. The new basaltic oceanic crust can gain Sr through interaction with seawater, but it is not a Sr donor. The subduction initiation and plate acceleration due to the increasing convergence velocities between Arabia and Eurasia during the Late Cretaceous could cause the uplift of the Arabian passive margin.

The Arabian Plate is characterized by a thick sequence of Cretaceous siliciclastic sedimentary rocks, which serve as a potential source for the significant influx of Rb and ^87^Sr/^86^Sr into the Neo-Tethys Ocean through uplift and erosion processes. The Dezful Embayment, located in the northwestern segment of the Persian Gulf, encompasses several distinct geological units, namely the Gadvan, Kazhdumi, Zubair, and Burgan siliciclastic units. Similar siliciclastic formations, such as the Zubair and Burgan formations, are also present in Saudi Arabia and Kuwait. These siliciclastic rocks likely underwent erosion during the uplift of the Arabian passive margin, concurrent with the deposition of the Sarvak Formation^30,73,74^.

Paleocurrent observations indicate that during the Cenomanian–Turonian period, the transport direction of siliciclastic sediment fluxes was from the southwest (encompassing the Arabian Plate including Saudi Arabia, Kuwait, and Iraq) towards the northeast (Dezful Embayment). This directional pattern strongly suggests the incorporation of siliciclastic sediments into the Sarvak Formation^36^.

This study shows that the subduction initiation not only controls the oceanic plate configuration, plate acceleration, or exhumation of the high-pressure rocks but also can cause the nearby continents to be uplifted and eroded. Although radiometric ages of magmatic and metamorphic rocks can provide insights into the timing of forearc formation and subduction initiation-related exhumation, the elemental and Sr isotope analysis of sedimentary sequences offers a more accurate means to determine the age of intra-oceanic thrusting and the construction of oceanic crust in conjunction with nearby continental landmasses.

Ophiolite obduction and intra-oceanic thrusting throughout the southeastern Neo-Tethys are believed to have occurred at different times along a west-to-east trajectory from Troodos (Cyprus), the Mediterranean (Turkey) to Oman, with the latter event occurring later^35^. Consequently, there is a proposal to analyze the strontium isotope composition of sedimentary sequences containing the studied disconformities across the Middle East in a west-to-east trajectory. This analysis aims to investigate whether the observed temporal pattern is reflected in absolute ages derived from strontium isotope ratios. Such an approach would help determine if strontium isotope analysis of sequences associated with regional disconformities offers a more precise method for defining the age of intra-oceanic thrusting and the formation of oceanic crust.

### Supplementary Information

Supplementary Information.

## Data Availability

All data generated and/or analyzed in this study are included in this published article and its supplementary information file, and are also available from the corresponding author on reasonable request.
