# Supplementary material for: Strontium isotope proxy of sedimentological records reveals uplift and erosion in the Southeastern Neo-Tethys ocean during the late Cretaceous
Source: Sci Rep. 2024 Feb 12;14:3499. doi: 10.1038/s41598-024-54128-3 (PMC10861479; doi:10.1038/s41598-024-54128-3)
Supplement: Supplementary file 1 — Supplementary Information. [file 41598_2024_54128_MOESM1_ESM.docx]

**Supplementary data**

**Table 1:** Sr and Rb elemental data along with un-corrected and corrected ^87^Sr/^86^Sr values for the studied cores AZ, MN, and GS-2.

| **Sample No.** | **Depth** | **87Sr/86Sr ratio** | **Std Err** | **[Rb] ppm** | **Sr (ppm)** | **Sr/Rb** | **Rb-corrected 87Sr/86Sr** | **Lower Sr values** | **Upper Sr Values** | **Lower age limit** | **Mean Age** | **Upper age limit** |
| --- | --- | --- | --- | --- | --- | --- | --- | --- | --- | --- | --- | --- |
| AZ 106 | 334.51 | 0.707469 | 0.000016 | 1 | 750 | 750 | 0.707464 | 0.707432 | 0.707496 | 85.6 | 82.84 | 81.41 |
| AZ 99 | 336.32 | 0.707457 | 0.000012 | 1 | 611 | 611 | 0.707451 | 0.707427 | 0.707475 | 85.85 | 83.64 | 82.9 |
| AZ 107 | 339.59 | 0.707448 | 0.000011 | 1 | 581 | 581 | 0.707441 | 0.707419 | 0.707463 | 86.17 | 84.75 | 82.9 |
| AZ 103 | 345.23 | 0.707472 | 0.00001 | 3 | 600 | 200 | 0.707453 | 0.707433 | 0.707473 |  |  |  |
| AZ 97 | 347.6 | 0.707425 | 0.000018 | 1 | 541 | 541 | 0.707418 | 0.707382 | 0.707454 |  |  |  |
| AZ 109 | 356.91 | 0.707453 | 0.000019 | 2 | 511 | 255.5 | 0.707438 | 0.7074 | 0.707476 | 86.79 | 85.12 | 82.2 |
| AZ 95.1 | 364.21 | 0.707462 | 0.000019 | 0 | 550 | #DIV/0! | 0.707462 | 0.707424 | 0.7075 | 85.98 | 82.95 | 80.8 |
| AZ 102 | 366.67 | 0.707453 | 0.00002 | 1 | 399 | 399 | 0.707443 | 0.707403 | 0.707483 | 86.7 | 84.45 | 81.79 |
| AZ 101 | 373.07 | 0.707442 | 0.000017 | 2 | 214 | 107 | 0.707406 | 0.707372 | 0.70744 |  |  |  |
| AZ 96 | 378.37 | 0.707428 | 0.000018 | 2 | 250 | 125 | 0.707397 | 0.707361 | 0.707433 |  |  |  |
| AZ 100 | 383.73 | 0.707422 | 0.000016 | 4 | 320 | 80 | 0.707374 | 0.707342 | 0.707406 |  |  |  |
| AZ 105 | 391.8 | 0.707421 | 0.000015 | 3 | 412 | 137.3 | 0.707393 | 0.707363 | 0.707423 |  |  |  |
| AZ 98 | 395.85 | 0.708006 | 0.000018 | 36 | 500 | 13.9 | 0.707729 | 0.707693 | 0.707765 |  |  |  |
| AZ 123 | 401.97 | 0.707955 | 0.000013 | 29 | 581 | 20.0 | 0.707763 | 0.707737 | 0.707789 |  |  |  |
| AZ 118 | 409.69 | 0.707812 | 0.000015 | 30 | 500 | 16.7 | 0.707581 | 0.707551 | 0.707611 |  |  |  |
| AZ 117 | 413.14 | 0.707826 | 0.000014 | 34 | 601 | 17.7 | 0.707609 | 0.707581 | 0.707637 |  |  |  |
| AZ 111 | 430.83 | 0.707265 | 0.000018 | 3 | 500 | 166.7 | 0.707242 | 0.707206 | 0.707278 |  |  |  |
| AZ 32 | 451.05 | 0.707265 | 0.00002 | 1 | 488 | 488 | 0.707257 | 0.707217 | 0.707297 | 89.6 | nd | nd |
| AZ 1 | 453.5 | 0.707301 | 0.000012 | 4 | 650 | 162.5 | 0.707277 | 0.707253 | 0.707301 |  |  |  |
| AZ 9 | 476.65 | 0.707242 | 0.000014 | 6 | 600 | 100 | 0.707204 | 0.707176 | 0.707232 |  |  |  |
| AZ 12 | 478.97 | 0.707282 | 0.000015 | 2 | 550 | 275 | 0.707268 | 0.707238 | 0.707298 | 89.58 | nd | nd |
| AZ 13 | 487.34 | 0.707234 | 0.000016 | 5 | 601 | 120.2 | 0.707202 | 0.70717 | 0.707234 |  |  |  |
| AZ 56 | 489.2 | 0.707265 | 0.000012 | 3 | 710 | 236.7 | 0.707249 | 0.707225 | 0.707273 | nd | nd | nd |
| AZ 14 | 490.26 | 0.70722 | 0.000018 | 4 | 719 | 179.8 | 0.707199 | 0.707163 | 0.707235 |  |  |  |
| AZ 29 | 493.01 | 0.707218 | 0.000019 | 2 | 800 | 400.0 | 0.707208 | 0.70717 | 0.707246 | nd | nd | nd |
| AZ 53 | 495.16 | 0.707245 | 0.000016 | 2 | 715 | 357.5 | 0.707234 | 0.707202 | 0.707266 | nd | nd | nd |
| AZ 26 | 497.1 | 0.707267 | 0.000016 | 1 | 649 | 649.0 | 0.707261 | 0.707229 | 0.707293 | 89.7 | nd | nd |
| AZ 15 | 500.3 | 0.707279 | 0.000019 | 1 | 600 | 600.0 | 0.707273 | 0.707235 | 0.707311 | 89.32 | nd | nd |
| AZ 31 | 503.55 | 0.70729 | 0.000013 | 1 | 400 | 400.0 | 0.70728 | 0.707254 | 0.707306 | 89.41 | nd | nd |
| AZ 51 | 504.01 | 0.707256 | 0.000016 | 6 | 601 | 100.2 | 0.707218 | 0.707186 | 0.70725 |  |  |  |
| AZ 33 | 504.57 | 0.707234 | 0.000016 | 7 | 700 | 100 | 0.707196 | 0.707164 | 0.707228 |  |  |  |
| AZ 27 | 506.46 | 0.707313 | 0.000017 | 2 | 450 | 225 | 0.707296 | 0.707262 | 0.70733 | 92.18 | 90.52 |  |
| AZ 40 | 507.64 | 0.707367 | 0.000016 | 3 | 700 | 233.3 | 0.707351 | 0.707319 | 0.707383 | 94.28 | 93.01 | 91.68 |
| AZ 83 | 508.25 | 0.707373 | 0.000015 | 0 | 900 | #DIV/0! | 0.707373 | 0.707343 | 0.707403 | 95.55 | 93.86 | 92.7 |
| AZ 22 | 508.48 | 0.707358 | 0.000013 | 7 | 500 | 71.4 | 0.707304 | 0.707278 | 0.70733 |  |  |  |
| AZ 70 | 508.86 | 0.707392 | 0.000015 | 4 | 450 | 112.5 | 0.707358 | 0.707328 | 0.707388 |  |  |  |
| AZ 79 | 509.31 | 0.707393 | 0.000019 | 6 | 950 | 158.3 | 0.707369 | 0.707331 | 0.707407 |  |  |  |
| AZ 54 | 511.28 | 0.707383 | 0.000017 | 3 | 800 | 266.7 | 0.707369 | 0.707335 | 0.707403 | 95.55 | 93.71 | 92.39 |
| AZ 58 | 511.91 | 0.707373 | 0.000015 | 2 | 800 | 400.0 | 0.707363 | 0.707333 | 0.707393 | 94.84 | 93.48 | 92.31 |
| AZ 44 | 513.53 | 0.708021 | 0.000015 | 29 | 550 | 19.0 | 0.707818 | 0.707788 | 0.707848 |  |  |  |
| AZ 48 | 514.04 | 0.708116 | 0.000014 | 32 | 750 | 23.4 | 0.707952 | 0.707924 | 0.70798 |  |  |  |
| AZ 42 | 519.83 | 0.708138 | 0.000019 | 25 | 650 | 26.0 | 0.70799 | 0.707952 | 0.708028 |  |  |  |
| AZ 49 | 520.44 | 0.707998 | 0.000017 | 23 | 500 | 21.7 | 0.707821 | 0.707787 | 0.707855 |  |  |  |
| AZ 19 | 523.9 | 0.707791 | 0.00002 | 27 | 540 | 20.0 | 0.707599 | 0.707559 | 0.707639 |  |  |  |
| AZ 69 | 534.46 | 0.707462 | 0.000019 | 4 | 471 | 117.8 | 0.707429 | 0.707391 | 0.707467 |  |  |  |
| AZ 77 | 535.98 | 0.707349 | 0.000016 | 1 | 365 | 365.0 | 0.707338 | 0.707306 | 0.70737 | 93.74 | 92.5 | 91.2 |
| AZ 80 | 540.5 | 0.707345 | 0.000014 | 4 | 250 | 62.5 | 0.707284 | 0.707256 | 0.707312 |  |  |  |
| AZ 68 | 542.84 | 0.707375 | 0.000019 | 3 | 240 | 80.0 | 0.707327 | 0.707289 | 0.707365 |  |  |  |
| AZ 74 | 544.21 | 0.707388 | 0.000017 | 2 | 320 | 160.0 | 0.707364 | 0.70733 | 0.707398 |  |  |  |
| AZ 78 | 545.13 | 0.707402 | 0.000015 | 2 | 518 | 259.0 | 0.707387 | 0.707357 | 0.707417 | 96.94 | 94.4 | 93.25 |
| AZ 92 | 556 | 0.707399 | 0.000015 | 0 | 651 | #DIV/0! | 0.707399 | 0.707369 | 0.707429 | 100.2 | 95.25 | 93.71 |
| AZ 90 | 558.11 | 0.707413 | 0.000014 | 1 | 641 | 641 | 0.707407 | 0.707379 | 0.707435 | nd | 95.7 | 94.1 |
| AZ 95 | 565.93 | 0.707433 | 0.000015 | 3 | 600 | 200 | 0.707414 | 0.707384 | 0.707444 | nd | 96.56 | 94.33 |
| AZ 59 | 567.93 | 0.707405 | 0.000018 | 1 | 500 | 500 | 0.707397 | 0.707361 | 0.707433 | nd | 95.11 | 93.4 |
| AZ 67 | 596.97 | 0.707435 | 0.000017 | 1 | 540 | 540 | 0.707428 | 0.707394 | 0.707462 | nd | 99.7 | 94.91 |
| AZ 60 | 600.87 | 0.707441 | 0.000019 | 2 | 400 | 200 | 0.707422 | 0.707384 | 0.70746 | nd | 97.79 | 94.33 |
|  |  |  |  |  |  |  |  |  |  |  |  |  |
| MN 69 | 592.78 | 0.707519 | 0.000014 | 22 | 750 | 34.1 | 0.707406 | 0.707378 | 0.707434 |  |  |  |
| MN 84 | 593.51 | 0.707511 | 0.000012 | 16 | 651 | 40.7 | 0.707417 | 0.707393 | 0.707441 |  |  |  |
| MN 81 | 597.1 | 0.707513 | 0.000008 | 24 | 440 | 18.3 | 0.707303 | 0.707287 | 0.707319 |  |  |  |
| MN 14 | 599.07 | 0.707462 | 0.000007 | 19 | 350 | 18.4 | 0.707253 | 0.707239 | 0.707267 |  |  |  |
| MN 74 | 600.71 | 0.707493 | 0.000015 | 25 | 487 | 19.5 | 0.707296 | 0.707266 | 0.707326 |  |  |  |
| MN 71 | 604.1 | 0.707473 | 0.000017 | 31 | 571 | 18.4 | 0.707264 | 0.70723 | 0.707298 |  |  |  |
| MN 37 | 607.51 | 0.707489 | 0.000017 | 20 | 590 | 29.5 | 0.707359 | 0.707325 | 0.707393 |  |  |  |
| MN 47 | 609.27 | 0.707482 | 0.000013 | 12 | 585 | 48.8 | 0.707403 | 0.707377 | 0.707429 |  |  |  |
| MN 23 | 611.7 | 0.707492 | 0.000015 | 15 | 587 | 39.1 | 0.707394 | 0.707364 | 0.707424 |  |  |  |
| MN 72 | 613.36 | 0.707806 | 0.000011 | 51 | 599 | 11.7 | 0.707479 | 0.707457 | 0.707501 |  |  |  |
| MN 95 | 616.07 | 0.707755 | 0.000014 | 45 | 500 | 11.1 | 0.707409 | 0.707381 | 0.707437 |  |  |  |
| MN 29 | 618.1 | 0.707512 | 0.000012 | 33 | 520 | 15.8 | 0.707268 | 0.707244 | 0.707292 |  |  |  |
| MN 96 | 624.49 | 0.707426 | 0.000015 | 11 | 545 | 49.5 | 0.707348 | 0.707318 | 0.707378 |  |  |  |
| MN 50 | 640.68 | 0.707444 | 0.000011 | 21 | 450 | 21.4 | 0.707265 | 0.707243 | 0.707287 |  |  |  |
| MN 39 | 643.02 | 0.707435 | 0.000013 | 2 | 461 | 230.5 | 0.707418 | 0.707392 | 0.707444 |  | 97.09 | 94.78 |
| MN 82 | 656.18 | 0.707439 | 0.000012 | 3 | 550 | 183.3 | 0.707418 | 0.707394 | 0.707442 |  |  |  |
| MN 15 | 657.88 | 0.707432 | 0.000013 | 11 | 551 | 50.1 | 0.707355 | 0.707329 | 0.707381 |  |  |  |
| MN 54 | 666.64 | 0.707466 | 0.000014 | 10 | 620 | 62.0 | 0.707404 | 0.707376 | 0.707432 |  |  |  |
| MN 42 | 667.79 | 0.707435 | 0.000009 | 4 | 590 | 147.5 | 0.707409 | 0.707391 | 0.707427 | 94.71 | 96.05 | 99.28 |
| MN 59 | 674.16 | 0.707423 | 0.000011 | 6 | 500 | 83.3 | 0.707377 | 0.707355 | 0.707399 |  |  |  |
| MN 24 | 676.49 | 0.707482 | 0.000013 | 9 | 490 | 54.4 | 0.707411 | 0.707385 | 0.707437 |  |  |  |
| MN 40 | 681.26 | 0.707421 | 0.000017 | 5 | 500 | 100.0 | 0.707383 | 0.707349 | 0.707417 |  |  |  |
| MN 34 | 684.96 | 0.707416 | 0.000017 | 9 | 650 | 72.2 | 0.707363 | 0.707329 | 0.707397 |  |  |  |
| MN 44 | 687.05 | 0.707511 | 0.000017 | 28 | 500 | 17.9 | 0.707296 | 0.707262 | 0.70733 |  |  |  |
| MN 1 | 692.81 | 0.70739 | 0.000013 | 2 | 550 | 275.0 | 0.707376 | 0.70735 | 0.707402 | 95.47 | 93.98 | 92.97 |
| MN 4 | 706.1 | 0.707326 | 0.000014 | 14 | 620 | 44.3 | 0.707239 | 0.707211 | 0.707267 |  |  |  |
| MN 43 | 721.75 | 0.707425 | 0.000014 | 17 | 450 | 26.5 | 0.70728 | 0.707252 | 0.707308 |  |  |  |
| MN 7 | 723.39 | 0.707721 | 0.000011 | 41 | 550 | 13.4 | 0.707434 | 0.707412 | 0.707456 |  |  |  |
| MN 73 | 725.2 | 0.707438 | 0.000011 | 24 | 600 | 25.0 | 0.707284 | 0.707262 | 0.707306 |  |  |  |
| MN 26 | 733.58 | 0.707325 | 0.000014 | 6 | 541 | 90.2 | 0.707282 | 0.707254 | 0.70731 |  |  |  |
| MN 30 | 739.48 | 0.707368 | 0.000014 | 7 | 400 | 57.1 | 0.707301 | 0.707273 | 0.707329 |  |  |  |
|  |  |  |  |  |  |  |  |  |  |  |  |  |
| GS-2 46 | 517.6 | 0.707499 | 0.000008 | 0 | 581 | #DIV/0! | 0.707499 | 0.707483 | 0.707515 | 81.79 | 80.87 | 79.48 |
| GS-2 47 | 523.14 | 0.707462 | 0.00001 | 5 | 750 | 150.0 | 0.707436 | 0.707416 | 0.707456 |  |  |  |
| GS-2 58 | 525.23 | 0.707469 | 0.000019 | 2 | 300 | 150.0 | 0.707443 | 0.707405 | 0.707481 |  |  |  |
| GS-2 48 | 527.34 | 0.707501 | 0.000013 | 11 | 400 | 36.4 | 0.707395 | 0.707369 | 0.707421 |  |  |  |
| GS-2 45 | 531.05 | 0.707478 | 0.000019 | 2 | 540 | 270.0 | 0.707464 | 0.707426 | 0.707502 | 85.9 | 82.84 | 80.64 |
| GS-2 56 | 538.95 | 0.707442 | 0.000019 | 6 | 433 | 72.2 | 0.707389 | 0.707351 | 0.707427 |  |  |  |
| GS-2 51 | 540.17 | 0.707433 | 0.000015 | 1 | 320 | 320.0 | 0.707421 | 0.707391 | 0.707451 | 87.05 | 86.09 | 83.64 |
| GS-2 59 | 546.95 | 0.707725 | 0.000018 | 23 | 260 | 11.3 | 0.707385 | 0.707349 | 0.707421 |  |  |  |
| GS-2 44 | 550.8 | 0.707365 | 0.000017 | 2 | 300 | 150.0 | 0.707339 | 0.707305 | 0.707373 |  |  |  |
| GS-2 43 | 552.58 | 0.707321 | 0.000016 | 0 | 600 | #DIV/0! | 0.707321 | 0.707289 | 0.707353 | 93.09 | 91.78 | 89.8 |
| GS-2 11 | 558.63 | 0.707355 | 0.000017 | 1 | 500 | 500.0 | 0.707347 | 0.707313 | 0.707381 | 94.19 | 92.85 | 91.38 |
| GS-2 1 | 559.42 | 0.707368 | 0.000014 | 3 | 581 | 193.7 | 0.707348 | 0.70732 | 0.707376 |  |  |  |
| GS-2 7 | 560.12 | 0.707805 | 0.000017 | 29 | 601 | 20.7 | 0.70762 | 0.707586 | 0.707654 |  |  |  |
| GS-2 12 | 560.64 | 0.707791 | 0.000015 | 21 | 650 | 31.0 | 0.707667 | 0.707637 | 0.707697 |  |  |  |
| GS-2 9 | 562.07 | 0.707771 | 0.000016 | 22 | 450 | 20.5 | 0.707583 | 0.707551 | 0.707615 |  |  |  |
| GS-2 15 | 568.77 | 0.707341 | 0.000014 | 3 | 450 | 150.0 | 0.707315 | 0.707287 | 0.707343 |  |  |  |
| GS-2 14 | 573.78 | 0.707353 | 0.000008 | 9 | 450 | 50.0 | 0.707276 | 0.70726 | 0.707292 |  |  |  |
| GS-2 32 | 578.42 | 0.707405 | 0.000013 | 10 | 321 | 32.1 | 0.707285 | 0.707259 | 0.707311 |  |  |  |
| GS-2 10 | 580.74 | 0.707392 | 0.000015 | 4 | 330 | 82.5 | 0.707345 | 0.707315 | 0.707375 |  |  |  |
| GS-2 20 | 587.72 | 0.707465 | 0.000016 | 6 | 400 | 66.7 | 0.707407 | 0.707375 | 0.707439 |  |  |  |
| GS-2 31 | 596.4 | 0.707425 | 0.000015 | 9 | 312 | 34.7 | 0.707314 | 0.707284 | 0.707344 |  |  |  |
| GS-2 30 | 599.27 | 0.707472 | 0.000017 | 5 | 251 | 50.2 | 0.707395 | 0.707361 | 0.707429 |  |  |  |

**References**

Allan, J.R., Wiggins, W.D., 1993. Dolomite Reservoirs: Geochemical Techniques for Evaluating Origin and Distribution. AAPG Short Course Note Ser. 36, 170.

Brand, U., Veizer, J., 1980. Chemical diagenesis of a multicomponent carbonate system - 1. Trace elements. Journal of Sedimentary Petrology 50, 1219-1236.

Faure, G., Mensing, T.M., 2004. Isotopes: principles and applications. John Wiley & Sons Inc.

Frijia, G., Parente, M., 2008. Strontium isotope stratigraphy in the upper Cenomanian shallow-water carbonates of the southern Apennines: Short-term perturbations of marine 87 Sr/86 Sr during the oceanic anoxic event 2. Palaeogeography, Palaeoclimatology, Palaeoecology 261, 15-29.

Gradstein, F.M., Ogg, J.G., Schmitz, M.D., Ogg, G.M., 2020. Geologic Time Scale 2020. Elsevier.

McArthur, J.M., 1994. Recent trends in strontium isotope stratigraphy. Terra Nova 6, 331-358.

McArthur, J.M., Howarth, R.J., 2004. Sr-isotope stratigraphy. A Geological Timescale 2004, 96-105.

McArthur, J.M., Howarth, R.J., Shields, G.A., 2012. Strontium Isotope Stratigraphy, The Geologic Time Scale 2012, pp. 127-144.

Mehrabi, H., Navidtalab, A., Enayati, A., Bagherpour, B., 2022a. Age, duration, and geochemical signatures of paleo-exposure events in Cenomanian–Santonian sequences (Sarvak and Ilam formations) in SW Iran: Insights from carbon and strontium isotopes chemostratigraphy. Sedimentary Geology 434, 106136.

Mehrabi, H., Navidtalab, A., Rahimpour-Bonab, H., Heimhofer, U., 2022b. Geochemical expression of sequence stratigraphic surfaces: A case from Upper Cretaceous shallow-water carbonates of southeastern Neo-Tethys margin, SW Iran. Cretaceous Research 140, 105329.

Navidtalab, A., Rahimpour-Bonab, H., Huck, S., Heimhofer, U., 2016. Elemental geochemistry and strontium-isotope stratigraphy of Cenomanian to Santonian neritic carbonates in the Zagros Basin, Iran. Sedimentary Geology 346, 35-48.

Ogg, J.G., Ogg, G., Gradstein, F.M., 2016. A concise geologic time scale: 2016. Elsevier.

Rahimpour-Bonab, H., Mehrabi, H., Navidtalab, A., Omidvar, M., Enayati-Bidgoli, A.H., Sonei, R., Sajjadi, F., Amiri-Bakhtyar, H., Arzani, N., Izadi-Mazidi, E., 2013. Palaeo-exposure surfaces in Cenomanian - Santonian carbonate reservoirs in the dezful embayment, SW Iran. Journal of Petroleum Geology 36, 335-362.

Steuber, T., Korbar, T., Jelaska, V., Gušić, I., 2005. Strontium-isotope stratigraphy of Upper Cretaceous platform carbonates of the island of Brač (Adriatic Sea, Croatia): Implications for global correlation of platform evolution and biostratigraphy. Cretaceous Research 26, 741-756.

Veizer, J., Ala, D., Azmy, K., Bruckschen, P., Buhl, D., Bruhn, F., Garden, G.A.F., Diener, A., Ebneth, S., Godderis, Y., Jasper, T., Korte, C., Pawellek, F., Podlaha, O.G., Strauss, H., 1999. 87Sr/86Sr, δ13C and δ18O evolution of Phanerozoic seawater. Chemical Geology 161, 59-88.
